# Plaster of Paris in the Treatment of Fractures

**Published:** 1889-03

**Authors:** W. F. Westmoreland

**Affiliations:** Atlanta, Ga.; Lecturer on Minor Surgery, Atlanta Medical College


THE ATLANTA MEDICAL AND SURGICAL JOURNAL.

VOL. VI. MARCH, 1889. NO. 1.

### Original Communications.

### PLASTER OF PARIS IN THE TREATMENT OF FRACTURES.

BY W. F. WESTMORELAND, JR., M. D.,

Lecturer on Minor Surgery, Atlanta Medical College.

For hundreds of years surgeons have been casting about, and instrument and fracture appliance makers have been taxing their ingenuity, for the purpose of devising some plan by means of bandages, splints, or fixtures, to hold the fragments of a fractured bone in their normal position, that union might properly take place, and thus prevent any deformity; so restoring the limb to its normal appearance and functions.

To mention the various devices, splints and appliances that have been suggested within the past century, would fatigue without instructing and interesting you. They are on record in magazines, medical journals, books on surgery and fractures, and we must refer you to them for further information on this subject.

The object of this paper is not to discuss the various appliances adopted by different surgeons, but to present to you what I regard the best plan; one that shows results in every particular more satisfactory to the surgeon and patient than any other plan that has been presented in the past or present. I allude to the plaster of Paris splint. It is not every plaster of Paris splint (so called) that is applied to a fractured bone that does well. It must be properly applied, of appropriate material, with the fragments of the fractured bone properly adjusted to give the results which are so much to be desired. For a time this splint was used only in simple fractures, but now, as will be seen later in this paper, we use them in compound comminuted fractures, with the very best results, often saving a life or a limb that was formerly sacrificed to the knife, or other plans of treatment that resulted in the death of the patient. To obtain the best results, it must be properly applied, and I will now give you the details of its application and the plan that I adopt. For simplicity, I have divided this paper into "Preparation of Material," "Treatment of Simple Fractures," and lastly, "Treatment of Compound Fractures."

### PREPARATION OF MATERIAL.

The bandage should be made out of ordinary cross-barred crinoline, that has had all the starch thoroughly washed out. The starch not only makes the crinoline very stiff and prevents its holding enough plaster, but also keeps the plaster from setting. Crinoline is better than gauze or cloth; the gauze pulls unevenly, and the meshes of the cloth are too fine to hold enough plaster to make a stout splint. Select the best plaster, and get it in unbroken cans; always test it by adding a little water to it, and see that it sets properly and quickly before using it. Put a small quantity of plaster on a table and rub it thoroughly into the meshes of the crinoline, and having an assistant to loosely roll the bandage as you rub. See that the plaster is evenly and smoothly distributed over the bandage. Rub the plaster into the bandage by hand. None of the machines that I have ever seen for this purpose, are worth the room they occupy. I prepare a large number of bandages at a time, having two or three widths, and pack them in a tin bucket, made air-tight. If placed in a dry place, they will keep for an indefinite length of time. Next, you will need flannel roller bandages, it will not do to use cotton under the plaster splint, as it is not comfortable to the patient, and is constantly kept damp by perspiration, and becomes offensive, and often excoriates the skin. I use a medium heavy red flannel for the bandages. Add to this a few pieces of perforated tin slips and a tape line and we are ready for the operation.

To better illustrate the application of a splint, we will take a case from practice. I receive a call that "Tom Jones had broken his leg. Come out at once and fix it up." "Get things ready to set a fractured leg," I call to my assistants. In five minutes the things are all packed in a satchel, and we are on our way to the patient. But pause a moment, and we will look at the contents of the satchel. It contains a bucket of plaster of Paris bandages, half a dozen flannel roller bandages, a pound of ether, a few narrow strips of perforated tin, a tape-line and a a pair of scissors. We reach the patient and find that Tom is a very stout, muscular man, weighing about two hundred pounds. An examination reveals an oblique fracture of the upper third of the right thigh, with two inches shortening. We immediately proceed to work; one assistant etherizes the patient, and the other gets things in order. An old sheet is put down by the bed to cover the carpet; on it, in easy reach, put a basin of cold water; place the plaster bandages close beside the basin, and have the flannel bandages at hand. While this is being done, I secure a few large books, tie them securely together, then wrap a towel around a broom-stick and fasten it. By this time Tom is etherized. I put the broom-stick down inside the side railing of the bed between two of the slats upright, while the end of the handle rests on the floor; an assistant holds it in this position. Now we pull Tom across the sides of the bed until his buttocks rest on the edge, while his legs hang over it, with the broom-handle between them, pressing against the perineum. I have Tom lifted, and put the book under his left buttock. This keeps him raised high enough for me to get around the pelvis with my bandage.

The assistant begins to make slow and steady extension; the counter-extension is made by the broom-handle, held firmly against the perineum. The towel wrapped around the broom-handle protects the perineum from injury. The slow, steady extension is kept up until the fracture is reduced; I hold both legs up side by side, and satisfy myself by exact measurement with the tape line, that both legs are the same length; keep up the extension all the time the bandage is being applied.

A flannel roll bandage is now applied, including the foot and extending up the whole length of the leg and around the pelvis, taking care that the bandage lies perfectly smooth, without any wrinkles.

I take a plaster bandage out of the basin where it has been previously placed, end upwards, with water enough to completely cover it, and squeeze out the *excess of water.* As the bandage is taken out of the basin, have a fresh one put in, and it will be ready by the time the former is used. The evidence that it is ready for use is that the bubbles of air have ceased to escape. The plaster bandage is smoothly applied over the flannel in the same manner that an ordinary roller would be, making the reverses whenever necessary. Do not pull the bandage, or try to exert pressure. Carry the bandages up the leg, including the foot and around the pelvis, going up as high in the perineum as possible. The idea is to fasten the leg immovably to the pelvis. As the bandages are applied, rub them thoroughly with the hand to squeeze out any air that may have gotten between the layers, and to rub the soft plaster into the meshes of each succeeding bandage. Make a number of extra turns around each joint, and work in a piece or two of perforated tin, letting them extend from below the knee to the crest of the illium, to strengthen the splint at those places, where the greatest strain exists.

There is no rule as to the number of layers to apply, but it must depend upon the character and seat of the fracture, and the muscularity of the patient.

Within a couple of hours of the receipt of the injury, the splint is applied, and when Tom recovers from the effects of the ether, he finds that he can turn in any direction, and occupy almost any position on the bed without pain. In fractures of the thigh, I do not let them out on crutches for the first week.

There are several points in the application of the plaster splint that I wish especially to emphasize. The most important is the immediate application of the splint. This has been bitterly opposed, by most of our book-makers especially. They, almost without exception, advocate waiting for a greater or less time until the swelling has subsided. What are the advantages of waiting? It is claimed that by waiting till the swelling subsides, the numerous accidents that are said to follow the immediate application, are to a certain extent eliminated.

If there was no other argument it suffices to say that the most of the men who advocate the plan of waiting, are the very men in whose hands these accidents occur, who report them, and who abandon this method with dissatisfaction and disgust. I think I understand this principle better than those with pre-conceived conclusions do. Experience is an excellent spy-glass; but it has this draw-back, that prejudice very often clouds the lens. One of our able book-makers is not even able to give personal experience as a reason for his prejudice, but he abuses and condemns this, as he does other splints, that he may better advocate his own.

His opinion, in this respect, reminds me of the founders of Lynn, Mass., who after exploring ten or fifteen miles, doubted whether the country was good for anything farther west than that.

There must be some reason for these failures. What are they? Surgeons who adopt the plan of waiting before applying the plaster splint, will resort to any temporary expedient to control the fractured bone. Unfortunately, the fracture is never controlled by these devices. The fragments are not held in apposition, the sharp ends of the fragments are continually piercing the soft parts with every movement of the patient, and every spasmodic movement of the muscles. The result of this continued irritation is inflammation and swelling. After a time, the swelling subsides sufficiently to suit the surgeon, and the plaster splint is applied. Later, and often before the bones unite, the swelling disappears—the splint being too loose to retain the fragments in position, the union is retarded, and when the bone at last unites there is often more or less deformity, and if it be an oblique fracture, the limb is shortened.

In the immediate application, the limb has not had time to swell, extension is kept up until the limb is enclosed in a splint that is accurately moulded to all the irregularities of the limb, both joints connected to the injured bone are fixed by the bandages being moulded into the bony prominences and depressions. This perfect adaptation of the splint to the inequalities of the limb absolutely prevents all motion, makes shortening an impossibility, and secures the complete immobility of the limb in a position in which it is put up. The fragments are held in perfect apposition. It is impossible for the ends of the broken bone to puncture and irritate the surrounding muscles. There are no spasms of muscles; no irritation; therefore it is impossible to have any inflammatory turgesence or swelling, and there is little danger of the splint getting tight. I have put up a fractured thigh where eight inches of the bone had been comminuted by a car wheel. Another, a compound fracture of both of the thighs by a street-car wheel. Another, fracture of the upper third of the thigh, with a fracture of the lower third of the same bone, with separation of the condyles. In neither of these was there any tightness, nor did the splints have to be removed, although in each case the splint was put on immediately after the injury. Watch the patient closely. Don't put on a splint and not see it for two or three days. Leave instructions that if the splint feels too tight, or the patient's foot or toes get cold or swell, to send for you; you may have to make many unnecessary visits as I have done, but in these cases it is better to make numerous unnecessary visits, than to fail to make the one that is necessary.

I always use an anæsthetic in the reduction of any fracture that requires considerable force. The principal cause of trouble in the displacement of fractures is the muscular contraction, and in reducing them, one main difficulty is to overcome this muscular action. No amount of extension or counter-extension will bring the fragments into position, much less retain them there, unless all muscular influence is removed. As this is true, the most natural way to overcome it is to etherize the patient. This does away with all possibility of having to use pulleys. The patient suffers no pain; is perfectly quiet, which is very important. There is no sudden contraction of the muscles at the wrong time. Never use pulleys to make extensions, or you will be apt to put up the limb in a bad position.

### COMPOUND FRACTURES.

No compound fracture by itself, no matter how extensive the comminution, is an indication for primary amputation, unless there is an accompanying irreparable destruction of the soft parts, including the large blood vessels. No doubt this seems an extreme principle to enunciate, but my experience in the treatment of compound fractures by this method, has convinced me that many limbs have been sacrificed to the surgeon's knife, which had they been left (on), would have been of infinite value to the patient. Nor is any argument needed to convince any one "especially the patient," that it is better to leave the limb in its natural position, than to remove it, and bury it forever out of sight. John Hunter said, two hundred years ago, "Surgery consists in curing a disease, rather than in a removal of it by mechanical means." But so differently do most people think on this subject, that a surgeon who performs the most operations, and gives most pain, is commonly thought the best.

When called to a compound fracture, I immediately etherize the patient, and am better able to reduce the fracture without muscular resistance, and retain it in perfect position, also to more thoroughly cleanse the wound.

Upon the thorough antiseptic cleansing of the wound and surrounding parts, and complete removal of all foreign and irritating matter, depends the quick and successful issue of the case, and in some instances even the life of the patient.

In no cases that we are called upon to treat, is a disregard of antiseptic details so inexcusable as in these. As soon as I see the patient, I cover the wound with a towel wrung out in bichloride sol. (1.500) and while the patient is being etherized, I cleanse the surrounding parts the same. When the patient is thoroughly etherized, I reduce the fracture, making the opening larger, if necessary for the easy replacing of the bones. Then I cleanse the wound, cutting the tissues until I can reach and touch every part of the wound with bi-chloride sol. For this I generally use (1.500) and afterwards wash it off with a weaker sol. (1.2000). (Sol. always means bi-chloride.) The instrument should be kept in carbolized sol. (1.30 or 40). All arteries should be ligated with cat-gut, and hemorrhages stopped and the wound closed with cat-gut sutures. Establish drainage either with rubber or bone drainage tubes, or cat-gut drains. Over the wound, place a thick layer of antiseptic gauze, and apply the flannel bandage, followed by the plaster of Paris as previously described. I am no advocate of heavy dressing under the splint, as I think this is sometimes the cause of accidents, by preventing the splint keeping the bones in apposition. I would rather cut a trap door in the splint and re-dress through it, as I have done successfully in a number of cases. I have been very much surprised that Scede's method of union has not been advocated in these cases, and I take this opportunity of advising it, especially in those where there is a great destruction of the soft parts. For as Gerster says, its simplicity and independence of the presence or absence of a sufficient covering of skin, commends it to the surgeon.

Scede's method consists in making use of the organizing power of the moist blood clots. He thus supplies the wound with a plastic material, rendering both drainage and compression unnecessary. Important arteries should be tied, the filling of the wound with blood being left exclusively to parenchymatous hemorrhages. When there is enough skin to close the wound, a small opening should be left at the upper angle to allow the drainage of superfluous blood; a piece of rubber tissue without any holes in it is placed over the wound, extending a small distance beyond its edge; this must lie closely and smoothly, and serves the double purpose of securing the complete filling of the wound with blood, and of preventing drying and evaporation, as well as keeping the bandage from absorbing any but superfluous blood. Then the rest of the dressing is the same as in other cases. It will be found that the blood in all parts of the wound will coagulate and will be gradually replaced without further secretion, by a permanent tissue.

Dennis, in *Medical News*, November 13th, 1886, reports 385 cases of compound fracture, with but one death, giving a mortality of less than one-third of one per cent. Previous to the adoption of antiseptic methods, the rate of mortality in the best of tables varied from twenty to sixty per cent.

